# Acteoside Binds to Caspase-3 and Exerts Neuroprotection in the Rotenone Rat Model of Parkinson's Disease

**DOI:** 10.1371/journal.pone.0162696

**Published:** 2016-09-15

**Authors:** Jiawen Yuan, Jinpeng Ren, Ying Wang, Xiao He, Yuwu Zhao

**Affiliations:** 1 Department of Neurology, Shanghai Sixth People's Hospital affiliated to Shanghai Jiao Tong University, Shanghai, China; 2 State Key Laboratory of Precision Spectroscopy and Department of Physics, East China Normal University, Shanghai, China; 3 NYU-ECNU Center for Computational Chemistry at NYU Shanghai, Shanghai, China; Suzhou University, CHINA

## Abstract

Parkinson’s disease (PD) is characterized by the progressive degeneration of the dopaminergic neurons in the substantia nigra (SN) region. Acteoside has displayed multiple biological functions. Its potential role against PD and the underlying signaling mechanisms are largely unknown. Here, we showed that oral administration of acteoside significantly attenuated parkinsonism symptoms in rotenone-induced PD rats. Further, acteoside inhibited rotenone-induced α-synuclein, caspase-3 upregulation and microtubule-associated protein 2 (MAP2) downregulation in PD rats. The molecular docking and molecular dynamics (MD) simulation results indicated that acteoside may directly bind to and inhibit caspase-3. Acteoside formed hydrogen bonds with at least six residues of caspase-3: ThrA177, SerA178, GlyA238, SerB339, ArgB341 and TrpB348. In addition, a pi-pi interaction was formed between acteoside and caspase-3’s HisA237, which might further stabilize the complex. MD simulation results demonstrated that the binding affinity of the caspase-3-acteoside complex was higher than that of caspase-3 and its native ligand inhibitor. Together, we show that acteoside binds to caspase-3 and exerts neuroprotection in the rotenone rat model of PD.

## Introduction

Parkinson’s disease (PD) is a common degenerative disorder in central nervous system (CNS) [1,2]. It is characterized by the progressive degeneration of the dopaminergic neurons of the substantia nigra (SN) [1,2]. The majority of PD cases will occur after the age of 50 [3], suggesting that the proportion of the patients with PD probably increase in the next decade [1,2,3]. The underlying molecular mechanisms of neuronal cell death in PD patients are still unclear [1,2], although the accumulation of α-synuclein in the Lewy bodies is the hallmark of PD [1,2].

The degeneration of dopaminergic neurons in SN is the pathological basis of the motor impairment in PD patient [1,2]. The altered expressions of multiple proteins, including α-synuclein [2], microtubule-associated protein 2 (MAP2) [4] and caspase-3 [5], would possibly cause proteasome dysfunction and mitochondrial impairment. These will lead to the subsequent loss of dopaminergic neurons [6,7]. Existing evidences have indicated that α-synuclein is involved in the regulation of dopamine release and transportation [7]. Soluble α-synuclein is primarily localized in the pre-synaptic regions of axons [7], which can form the filamentous aggregates [7]. The latter is the major non-amyloid components of intracellular inclusions in several neurodegenerative diseases [7]. The insoluble and aggregated α-synuclein will induce the fibrillization of microtubule-associated protein tau [7] to kill the neuronal cells. MAP2 is a member of microtubule-associated protein family, which stabilizes microtubules against de-polymerization [8,9]. MAP2 is also a late neuronal marker, and its expression is important toward neuronal survival [8,9]. Increased expression of MAP2 will lead to expression of anti-apoptotic molecules and decrease in caspase-3 activation [8,9].

Caspase-3 is a primary apoptosis-related cysteine peptidase that is critical for the activation of caspase cascade process [10,11]. Caspase-3 is activated in the response to oxidative stress via the proteolytic cleaving by poly (ADP-ribose) polymerase (PARP) [11]. The cleavage will lead to the activation of the sterol regulatory element binding proteins (SREBPs) between the basic helix-loop-helix leucine zipper domain and the membrane attachment domain [11]. Multiple studies have demonstrated that inhibition or in-activation of caspase-3 can protect neurons from a number of stimuli [5,12]. Caspase-3 inhibition could also protect dopaminergic neurons from multiple stressors [5,12].

Acteoside is a member of the dihexose family [13]. It is the main hydroxycinnamic derivative in many species, including bitter gourds and olives [13]. Studies have indicated that acteoside could exhibit anti-allergic, anti-inflammatory and anti-proliferative functions [13]. Recent studies have also indicated that acteoside could exhibit neuroprotective activities [14,15,16,17,18,19]. For example, acteoside was shown to inhibit the neuronal death induced by 1-methyl-4-phenylpyridinium ions (MPP^+^) and glutamate [18,19]. It could also protect human neuroblastoma SH-SY5Y cells against β-amyloid [17].

In the present study, we first investigated the potential neuron-protective effect of acteoside in a rotenone-induced PD rat model. The underlying signal mechanisms were also studied though testing PD-associated proteins, including α-synuclein, caspase-3 and MAP2 [8]. Our results showed that acteoside attenuated neuronal damage in the PD rats. Further molecular dynamics studies revealed that acteoside may directly interact with caspase-3, thus inhibiting neuronal cell apoptosis.

## Materials and Methods

### Rotenone-induced PD rat model

The Sprague Dawley (SD) rats, weighing 200~250 g, were purchased from the laboratory of animal science institute of Fudan University (Shanghai, China). The rats were randomly divided into three groups: control, rotenone model (“model”) or rotenone plus acteoside treatment (“treatment”), with 10 rats per group. The latter two groups, “model” and “treatment”, received rotenone injections. Rotenone (Sigma, Shanghai, China) was initially dissolved in dimethyl sulphoxide (DMSO, 0.1%, Sigma) and then in olive oil (0.2 mg/mL). Subcutaneous rotenone injection (1 mg/kg/d) was performed in the “model” and “treatment” rats for 42 consecutive days. For the “treatment” group, acteoside (30 mg/kg/d, oral gavage, for 42 days) was also administrated. The control group was injected with the same amount of olive oil (vehicle). The clinical signs of rats were observed on daily basis, and if the criteria of humane endpoints were met, animals were sacrificed. Humane endpoints were considered as rapid weight loss (>15%), severe fever, vomiting or skin problems (wounds or signs of inflammation). If animals reached these endpoints they were euthanized by exsanguination under 2,2,2-tribromoethanol anesthesia (4 mg/10 g body weight, Sigma). All injections in this study were performed via the 2,2,2-tribromoethanol anesthesia method. The animal protocol was approved by the Institutional Animal Care and Use Committee (IACUC) and Ethics Review Board (ERB) of Shanghai Jiao Tong University.

### Behavioral test

The open field test (OFT) was applied to evaluate the rats’ behaviors [20,21]. The PD model rats were placed in the central area of the open field system and recorded for 5 min. The observed indicators included the following: (1) the number of times the rats entered into the adjacent area, (2) the duration in which the forelimbs were off the ground by more than 1 cm, and (3) the duration that rats stayed in the central area. Additionally, gait instability, muscle tremors, slowed activity and other abnormal behaviors were also observed.

### Immunohistochemistry (IHC) staining

The IHC staining was performed based on the protocol described in previous study [22]. The glyceraldehyde-3-phosphate dehydrogenase (GAPDH, sc-365062), α-synuclein (sc-12767) and MAP-2 (sc-74422) antibodies were purchased from Santa Cruz Biotech (Santa Cruz, CA).

### Western blot

The Western blot protocol was performed as described by Kubli et al [23]. The intensity of each protein band (in total gray) was quantified via the ImageJ software (NIH, Bethesda, MD), the value was normalized to each equal loading (GAPDH).

### Statistical analysis

All data were expressed as the means ±SD. The statistical analyses were performed using SPSS version 18.0 (Chicago, CA). Student’s t-tests were applied to evaluate differences between two groups, and one-way ANOVA tests were utilized to test the differences between multiple groups. A P-value < 0.05 was considered as significant difference.

### Molecular docking

The high-resolution crystal structure of caspase-3 was downloaded from Protein Data Bank (id: 1RHQ). The docking of the native ligand and acteoside was performed using Glide software [24]. The protein was prepared using the Protein Preparation Wizard module [24]. All of the crystallographic water molecules were removed. Hydrogen atoms were added using an Amber ff99SB force field [24]. A docking protocol file was generated via Receptor Grid Generation module, and the active site was confirmed with reference to the native ligand. The ligand was prepared using the Ligprep option. The binding site was located on the same site as the reference ligand, which was the inhibitor binding site of caspase-3. The native ligand for docking was extracted from the complex structure retrieved from the Protein Data Bank. Acteoside molecule file was downloaded from the NCBI website. The composition with the lowest score was saved for further study.

### Molecular dynamics (MD) simulation

Hydrogen atoms were added to target protein with the TLEAP module based on the Amber force field (ff99SB [25]) of Amber 12 [26]. The geometry of acteoside was optimized at the HF/6-31G* level [25]. Subsequently, the force field parameters of the ligand were obtained using the ANTECHAMBER module based on the Generalized Amber force field (GAFF) with the HF/6-31G* RESP charges [27,28]. This complex was then soaked in a TIP3P water box with 10 Å buffer. A 10, 000 step minimization was performed with a quadratic constraint on the residues of caspase-3 with a force constant k = 500 kcal/(mol•Å^2^), followed by a 30,000 step minimization without any restraints. Subsequently, the whole system was heated to 300 K in 10 ps with a weak restraint force constant of k = 10 kcal/(mol•Å^2^) on caspase-3 with a time step of 1 fs. Finally, a 40 ns simulation with a time step of 2 fs was performed on the complex at 300 K. All minimization and MD simulation were performed using Amber 12. The SHAKE algorithm was utilized to constrain all of the chemical bonds involving hydrogen atoms. For the long-range electrostatic interactions, the particle mesh Ewald (PME) method was used, and a standard 10 Å cutoff was used for the van der Waals interactions. Langevin dynamics with a collision frequency of 1.0 ps^-1^ were applied to control the temperature. The configurations were collected every 1 ps.

### MM/PBSA calculation

The binding free energy of the caspase-3-ligand complex was calculated using molecular mechanics/Poisson−Boltzmann surface area (MM/PBSA) approach. Each decomposed energy term was computed as follows:

$$\Delta G={\Delta E}_{elec}+{\Delta E}_{vdw}+{\Delta G}_{PB}+{\Delta G}_{nonpolar}-T\Delta S$$

Where ΔE_elec_ and ΔE_vdw_ are the electrostatics and van der Waals energies between the protein and ligand, respectively, ΔG_PB_ and ΔG_nonpolar_ are the polar and nonpolar components of the desolvation energy, respectively, and TΔS is the change in the conformational entropy upon ligand binding. In the MM/PBSA calculation, the value of the exterior dielectric constant was set to 80, and the solute dielectric constant was set to 1. The salt concentration was set to 0.1 mol/L. The nonpolar solvation term was calculated from the solvent-accessible surface area (SASA) [29] using the MSMS program as follows: ΔG_nonpolar_ = γ × ΔSASA [where γ = 0.0072 kcal/(mol•Å2), and the unit of ΔSASA is Å^2^. In this study, the change in the conformational entropy was not included in MM/PBSA calculations because of the large fluctuation and tremendous computational cost for the calculation of this term.

## Results

### Acteoside attenuates parkinsonism symptoms in rotenone rats

Tremor paralysis is a typical symptom of parkinsonism [20]. Starting from one week of daily rotenone injection, symptoms of PD were evident. The majority of the rats exhibited increases in saliva secretion, piloerection, hypotonia, respiratory frequency, as well as sensitivity to environmental stimuli and eating difficulties (Data not shown). The OFT test revealed that the rotenone PD rats exhibited significantly increased time in the central area (P<0.05), along with reduced movement (P<0.05) and standing times (P<0.01) (Table 1). Remarkably, such parkinsonism symptoms were largely alleviated with co-administration of acteoside (Table 1). Therefore, oral administration of acteoside attenuates parkinsonism symptoms in rotenone-induced PD rats.

**Table 1:** OFT test results.

Group	N	Stay in the central area (seconds)	Area Cross (times/5 min)	Standing up (times/5 min)	Standing up (seconds)
Control	10	6.3±3.1	31.0±10.6	42.0±16.6	16.6±7.2
PD	10	16.6±7.3^a^	9.1±4.2^a^	3.6±2.6^b^	3.1±2.2^a^
Treatment	10	11.3±2.6^c^	12.1±6.5^c^	9.8±5.9^c^	7.5±5.1^c^

($\bar{x}$±s)

^a^ *P*<0.05 (*vs*. “Control”)

^b^ *P*<0.01 (*vs*. “Control”)

^c^ *P*<0.05 (*vs*. “PD”).

### Acteoside attenuates rotenone-induced α-synuclein, caspase-3 upregulation and MAP2 downregulation in rats

**Fig 1:**
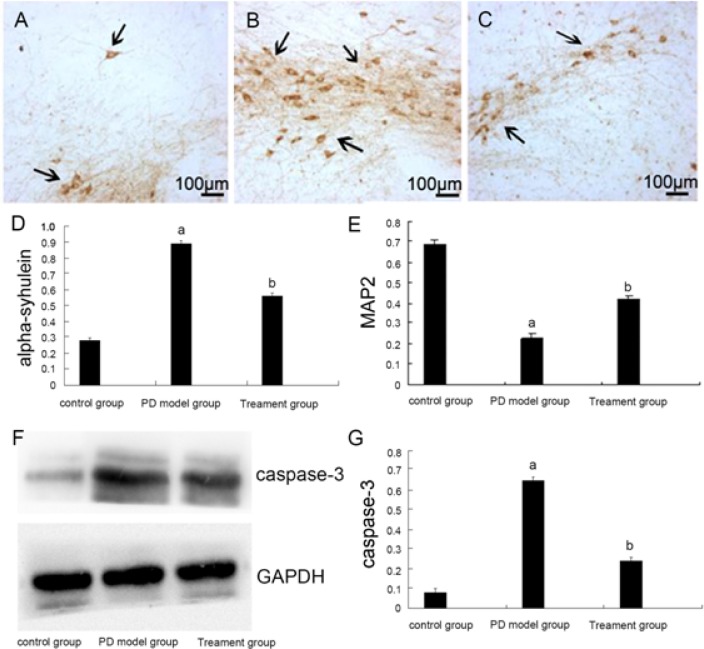
Acteoside attenuates rotenone-induced α-synuclein, caspase-3 upregulation and MAP2 downregulation in rats. Immunohistochemistry (IHC) for α-synuclein in A) the control group, B) the rotenone PD model group and C) plus acteoside treatment group. D) The expression levels of α-synuclein. a: P<0.01 compared to control and b: P<0.01 compared to the model. E) Expression levels of MAP2. F) Western blot assay for caspase-3. G) Expression levels of caspase-3 in the control, PD model and acteoside treatment (“Treatment”) groups: a: P<0.01 compare to control and b: P<0.01 compare to PD model.

The expression profiles of three key PD-associated proteins, including α-synuclein [2], MAP2 [4] and caspase-3 [5], were tested in the rats. The IHC results demonstrated that expression of α-synuclein was significantly increased in rotenone rats’ substantia nigra (SN) regions (Fig 1). Further, caspase-3 level, tested by Western blot assay, was also increased in SN regions of rotenone rats (Fig 1). On the other hand, MAP2 IHC intensity was decreased in the PD rats (Fig 1, same SN regions). Dramatically, such signaling changes (α-synuclein, caspase-3 upregulation and MAP2 downregulation) were largely attenuated with co-administration of acteoside (Fig 1). Thus, acteoside administration attenuates rotenone-induced altered expression of α-synuclein, caspase-3 and MAP2 in rats.

### Acteoside targets caspase-3

**Fig 2:**
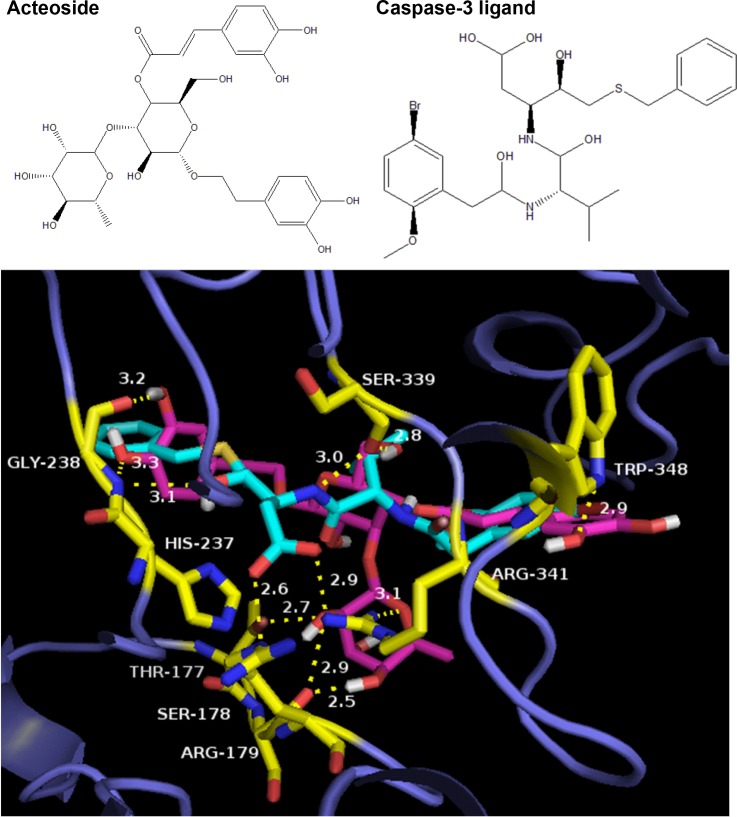
The binding between caspase-3 with acteoside or its the crystallized ligand. Upper: the molecular structure of acteoside (left) and the crystallized ligand (right). Lower: the docked complex structure of caspase-3 and acteoside with reference to the native ligand. The protein is shown in blue. Acteoside is in pink and the native ligand is in cyan. The residues forming strong interactions are in yellow. The yellow dashed lines represent the hydrogen bonds between the protein residues and bound ligands. The numbers beside the dashed lines are the distance (in Å) between the two heavy atoms of the hydrogen bonds.

**Fig 3:**
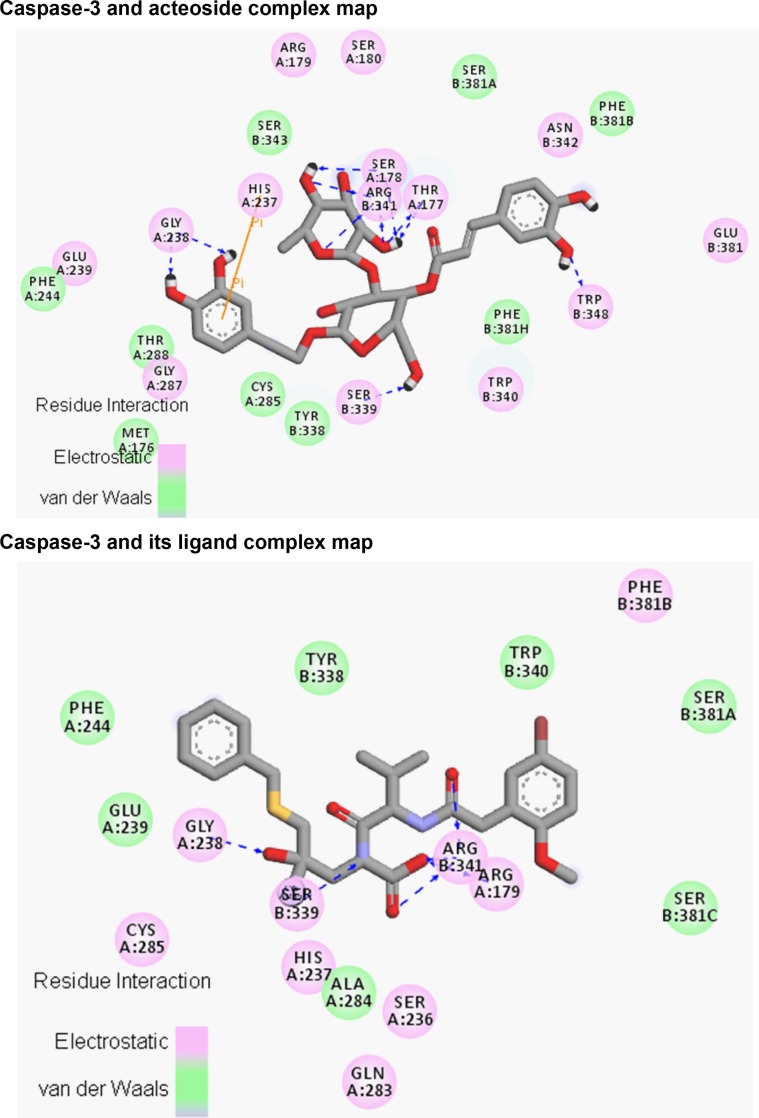
The 2D interaction map of caspase-3 and acteoside (or ligand) complexation. Upper: the interaction map between caspase-3 and acteoside. Lower: the interaction map between caspase-3 and its crystallized ligand. The pink and green balls represent the residues around the molecule. The blue dashed lines denote hydrogen bonds.

Above results demonstrated that acteoside significantly inhibited rotenone-induced α-synuclein and caspase-3 expression in rats. Therefore, it is possible that these two are direct target proteins of acteoside. The potential binding between caspase-3 and acteoside in several hydrogen bonds was demonstrated by molecular docking and verified in the molecular dynamics (MD) simulation method (Figs 2 and 3). The results indicated that caspase-3 could be a potential target of acteoside (See results bellow). On the other hand, using the similar methods, we failed to observe apparent binding between acteoside and α-synuclein (Data not shown). Therefore, acteoside could directly target caspase-3 (See results below).

### Docking results and binding properties of the caspase-3-acteoside complex

The acteoside molecule was docked to caspase-3 protein using the Glide software. The binding site is located at the same site of the crystallized ligand, which is a ligand inhibitor of caspase-3 [30]. The docking score (GlideXP) of the acteoside molecule was -7.78, while the docking score of the crystallized ligand was -7.37, indicating that the binding between caspase-3 and acteoside was even more stronger than that with the original ligand [30] (Fig 2). The docked complex structure of caspase-3 with acteoside was shown in Fig 2, with reference to the crystallized ligand (Fig 2). The structure was drawn using the Pymol program. The 2D interaction maps of the complexes (generated using Discovery Studio 3.5) were shown in Fig 3. Eleven hydrogen bonds were formed between acteoside and caspase-3, including the following six residues: ThrA177, SerA178, GlyA238, SerB339, ArgB341 and TrpB348 (Fig 3).

Significantly, There was also a pi-pi interaction between acteoside and residue HisA237 of caspase-3 (Fig 3). The native ligand formed hydrogen bonds with only four residues of caspase-3 (Fig 3). Three of them also formed hydrogen bonds with acteoside, including GlyA238, SerB339 and ArgB341 (Fig 3). Another hydrogen bond was formed between the native ligand and residue ArgA179 of caspase-3 (Fig 3). The formation of these bonds between acteoside and caspase-3 could stabilize the binding, therefore influencing the biological activities of caspase-3. There were also electrostatic interactions between acteoside and caspase-3 (Fig 3), possibly due to the fact that acteoside contains many polar oxygen atoms with high electron-withdrawing abilities. These interactions might also be responsible for the formation of the numerous hydrogen bonds during the formation of the caspase-3-acteoside complex (Fig 3).

### Stabilities and binding free energies of caspase-3-acteoside complex

**Fig 4:**
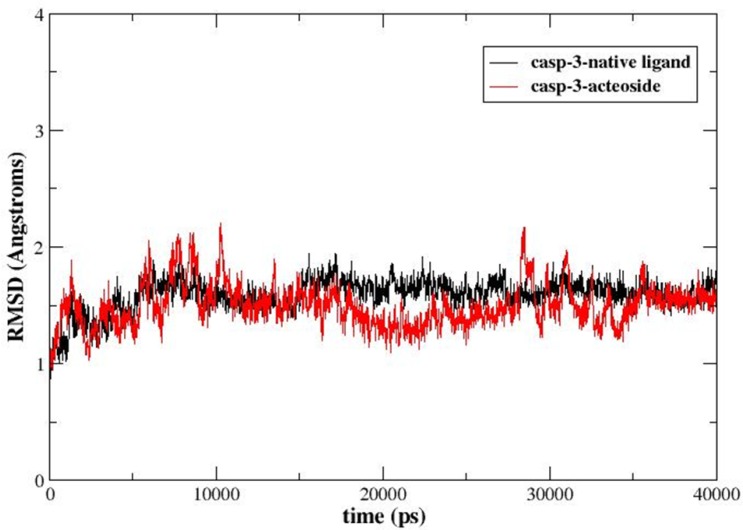
The evolution of the backbone root-mean-square deviation (RMSD) of the caspase-3-acteoside (or ligand) complex. The black curve represents the RMSD of the complex for the native ligand bound to caspase-3, and the red curve denotes the RMSD of the caspase-3-acteoside binding complex.

The caspase-3-acteoside complex with the highest score in Glide docking results was saved. Subsequently, MD simulation and molecular mechanics/Poisson−Boltzmann surface area (MM/PBSA) calculations were performed. The MD simulation was performed for 40 ns. Fig 4 demonstrated the evolution of backbone root-mean-square deviation (RMSD) of the complex with respect to the minimized structure over time. MD simulation of the binding complex of caspase-3 and native ligand was also carried out for 40 ns for comparison. As shown in Fig 4, the RMSDs of backbone atoms for these two binding complexes were mostly below 2 Å, indicating that the overall binding structures were indeed very stable during 40 ns MD simulation.

Fifty snapshots were taken at 40 ps intervals of the most stable 2 ns trajectory and extracted for the MM/PBSA calculations. As shown in Table 2, the total binding affinity between caspase-3 and acteoside was -32.76 kcal/mol, again indicating that the binding of the complex is stable. The binding energy of caspase-3 with its native ligand was -25.98 kcal/mol (Table 2). Therefore, based on these calculations, we suggest that caspase-3 binds to acteoside with a higher affinity than its crystallized ligand. For both ΔE_elec_ and ΔE_vdw_ analysis, the interaction energies between acteoside and caspase-3 were much higher than that of the native ligand (Table 2). Both the electrostatic interactions and the van der Waals interactions could play important roles in molecular binding between acteoside and caspase-3. Moreover, several hydrogen bonds and other electrostatic and van der Waals interactions were also formed between acteoside and caspase-3, which could possibly lead to even stronger binding of this complex.

**Table 2:** The binding free energies (kcal/mol) between the caspase-3 and the acteoside calculated by the MM/PBSA method. a) “STD” stands for standard deviation.

		ΔE_elec_	ΔE_vdw_	ΔG_nonpolar_	ΔG_PB_	ΔE_elec_+ΔG_PB_	ΔG_PB_
Caspase-3- Acteoside	Energy (kcal/mol)	-79.52	-62.69	-5.97	115.41	35.89	-32.76
	STD^a^	8.35	4.21	0.17	6.16	7.64	6.26
Caspase-3- native ligand	Energy (kcal/mol)	-58.59	-58.55	-4.43	95.59	37.00	-25.98
	STD^a^	8.76	4.99	0.18	7.80	3.85	5.47

**Fig 5:**
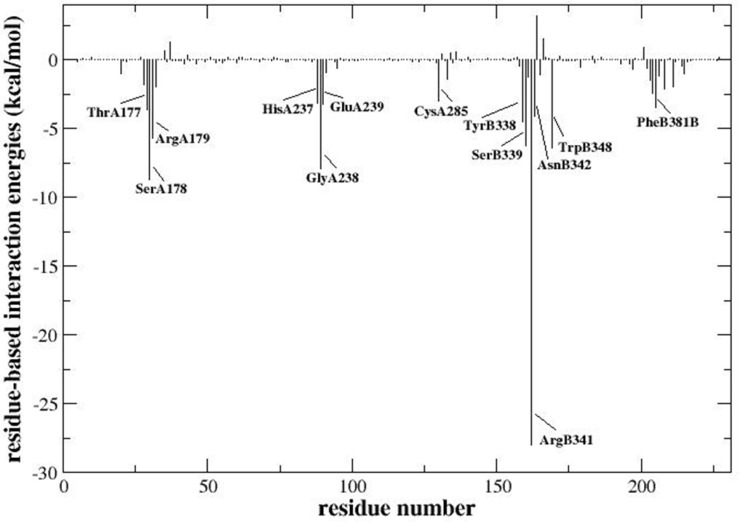
The interaction energy between each residue of caspase-3 and acteoside. Only electrostatic interactions and van der Waals interactions were included.

The sum of the electrostatic and van der Waals interaction energies between each residue of caspase-3 and acteoside was listed in Fig 5. The relative strengths of the interaction energies between the molecule and each residue were also compared. The relatively stronger interactions (below -2 kcal/mol) were shown in Table 3. The residues ThrA177, SerA178, GlyA238, SerB339, ArgB341 and TrpB348, which formed hydrogen bonds with acteoside, exhibited strong interactions with the molecule (Fig 5 and Table 3). Especially, ArgB341 had strongest interaction with acteoside, due to three hydrogen bonds formed between ArgB341 and different locations of acteoside (Fig 5 and Table 3). GlyA238 and SerA178 both formed two hydrogen bonds with acteoside, also showing strong binding interaction energies (Fig 5 and Table 3). In addition, HisA237, which formed a pi-pi interaction with acteoside, had relatively large van der Waals interaction of -3.42 kcal/mol (Fig 5 and Table 3). There were also other residues that formed strong interactions with acteoside, all of which were located in the binding pocket and likely provided the major contribution to molecular binding of this complex (Fig 5 and Table 3).

**Table 3:** The electrostatic interaction energies, van der Waals interaction energies and sums of these energies (kcal/mol) between acteoside and several key residues that form strong interactions or hydrogen bonds with caspase-3 (the interaction energies were stronger than -2.0 kcal/mol).

Residues	ΔE_elec_	ΔE_vdw_	ΔE_elec_+ΔE_vdw_
ThrA177	-2.64	-1.00	-3.64
SerA178	-11.9	3.21	-8.70
ArgA179	-4.13	-1.57	-5.70
HisA237	0.30	-3.42	-3.12
GlyA238	-6.48	-1.41	-7.89
GluA239	-1.71	-1.54	-3.25
CysA285	-0.75	-2.22	-2.97
TyrB338	0.30	-4.84	-4.54
SerB339	-5.80	-0.43	-6.24
ArgB341	-24.12	-3.84	-27.97
AsnB342	-1.55	-2.54	-4.09
TrpB348	-4.79	-1.62	-6.41
SerB381	-0.52	-1.92	-2.44
PheB381	-1.63	-1.84	-3.47
AspB381	-1.97	-0.16	-2.13

## Discussions

The loss of dopaminergic neurons in the SN is known as the primary pathological cause of PD [6]. The abnormal expression of α-synuclein has been shown to be strongly associated with dopaminergic neuronal cell death [1,2,6,7]. α-synuclein aggregation in dopaminergic neurons will cause downregulation of pro-survival MAP2 [8,9] and increased expression of caspase-3 [5,9]. These changes will likely induce mitochondrial depolarization and dopaminergic neuron cell apoptosis [1,2,5,6,7,8,9].

Acteoside has displayed certain neuroprotective activity and could protect neuronal cells from MPP^+^ and glutamate challenges [18,19]. In the present report, we showed that acteoside significantly relieved the symptoms of rotenone-induced parkinsonism. Moreover, we measured the expressions of three PD-associated proteins, including α-synuclein, MAP2 and caspase-3. Our results showed that acteoside oral administration significantly decreased the expressions of α-synuclein and caspase-3, but elevated the expression of MAP2 in the SN regions of the rotenone PD rats. These signaling changes could be the reason of its neuroprotective effect against parkinsonism symptoms in rotenone-induced PD rats.

Significantly, our molecular docking and MD simulation results showed that acteoside may directly bind to caspase-3 (but not α-synuclein) and inhibit its function. Acteoside binding to caspase-3 possibly inhibited neuronal cell apoptosis by rotenone. We showed that acteoside formed hydrogen bonds with at least six residues of caspase-3. Furthermore, a pi-pi interaction was also formed between acteoside and caspase-3’s HisA237. The MD simulation studies revealed that the binding affinity of the caspase-3-acteoside complex was even higher than that of caspase-3 with its native ligand inhibitor.

## Conclusions

In summary, acteoside protects against parkinsonism symptoms in rotenone-induced PD rats possibly via directly binding to caspase-3. The results may provide valuable insights in rational drug design of novel and potent caspase-3 inhibitors for PD treatment.
